# Rapid and robust squashed spore/colony PCR of industrially important fungi

**DOI:** 10.1186/s40694-023-00163-0

**Published:** 2023-07-08

**Authors:** Guoliang Yuan, Jeffrey J. Czajka, Ziyu Dai, Dehong Hu, Kyle R. Pomraning, Beth A. Hofstad, Joonhoon Kim, Ana L. Robles, Shuang Deng, Jon K. Magnuson

**Affiliations:** 1 Chemical and Biological Processes Development Group, Pacific Northwest National Laboratory, Richland, WA 99352 USA; 2 DOE Agile BioFoundry, Emeryville, CA 94608 USA

**Keywords:** Spore PCR, Colony PCR, Squash preparation, Spore concentration, Quick screening

## Abstract

**Background:**

Fungi have been utilized for centuries in medical, agricultural, and industrial applications. Development of systems biology techniques has enabled the design and metabolic engineering of these fungi to produce novel fuels, chemicals, and enzymes from renewable feedstocks. Many genetic tools have been developed for manipulating the genome and creating mutants rapidly. However, screening and confirmation of transformants remain an inefficient step within the design, build, test, and learn cycle in many industrial fungi because extracting fungal genomic DNA is laborious, time-consuming, and involves toxic chemicals.

**Results:**

In this study we developed a rapid and robust technique called “Squash-PCR” to break open the spores and release fungal genomic DNA as a template for PCR. The efficacy of Squash-PCR was investigated in eleven different filamentous fungal strains. Clean PCR products with high yields were achieved in all tested fungi. Spore age and type of DNA polymerase did not affect the efficiency of Squash-PCR. However, spore concentration was found to be the crucial factor for Squash-PCR in *Aspergillus niger*, with the dilution of starting material often resulting in higher PCR product yield. We then further evaluated the applicability of the squashing procedure for nine different yeast strains. We found that Squash-PCR can be used to improve the quality and yield of colony PCR in comparison to direct colony PCR in the tested yeast strains.

**Conclusion:**

The developed technique will enhance the efficiency of screening transformants and accelerate genetic engineering in filamentous fungi and yeast.

**Supplementary Information:**

The online version contains supplementary material available at 10.1186/s40694-023-00163-0.

## Background

Microorganisms such as fungi and yeast can produce a wide variety of valuable compounds that are used in various industries, including in food, pharmaceutical, and chemical processes [1–4]. These biobased compounds are attractive alternatives to traditional petroleum-based manufacturing as they can help reduce our reliance on fossil fuels and can alleviate some environmental impacts. Genetic engineering of microorganisms is the most effective means to enhance host productivity of natural compounds and can also be used to introduce new biosynthetic pathways to microbes allowing for the production of non-native compounds. Recent advances in synthetic biology tools and methods have significantly enhanced the throughput of microbial engineering. In parallel, improved multi-omic data collection and analysis pipelines have enabled a more comprehensive understanding of the underlying biology of targeted hosts, leading to more effective genetic engineering target selections. These advancements in synthetic biology and omic analyses have led to an exponential increase in the number of engineered microbial strains, which require rapid genotype screening to verify that the constructed strain is correct.

PCR remains the most common method for verifying DNA edits. Conventional PCR screening utilizes genomic DNA from the genetically modified organism as a template for amplification. Current protocols typically require the cultivation of the modified cell to generate enough biomass for genomic DNA extraction. Mechanical techniques such as bead-beating or mortar/pestle grinding are most commonly used to disrupt cell walls [5, 6]. The genomic DNA is then isolated by a traditional genome extraction protocol that requires toxic reagents (i.e., phenol) and often takes several hours. Overall, the whole procedure usually requires several days to complete the PCR verification, and it is challenging to implement in a high throughput manner. For bacteria, it is possible to skip the biomass cultivation step by using PCR directly on the transformed colony (colony PCR) instead of isolated genomic DNA [7]. However, there is no comparable method for fungi as the tough and complex outer layers of fungal spores and some yeast cells significantly reduce the extraction of free genomic DNA for an effective PCR [8, 9]. As a result, developing a reliable PCR method directly from spores (spore PCR) or yeast cells has been problematic.

The vital step of colony or spore PCR is to disrupt the intact cells to release the genomic DNA into the extracellular media. Common methods for mechanical cell disruptions such as bead milling or grinding with a mortar and pestle are not reliably applicable to fungal spores as the fungal spores are several micrometers in diameter and cannot be disrupted effectively due to the complex spore layers. Some spore PCR methods have been developed with success in a limited number of species. For example, Xu and Hamer successfully performed spore PCR in *Magnaporthe grisea* by freezing the samples and found that an excessive number of spores could inhibit the PCR reaction [10]. Subsequently, Ferreira et al. described a quick template preparation method by microwave irradiation for PCR amplification with *Neurospora crassa* spores [8]. AlShahni et al. reported that an Ampdirect® Plus kit allows direct PCR amplification from spores with no requirement for DNA extraction in *Penicillium expansum* TIMM 1293, *Aspergillus unguis* JCM 2256 and *A*. *sclerotiorum* JCM 1962 [11]. More recently, Fraczek et al. utilized a thermal-shock method to release spore genomic DNA for PCR [9]. While these techniques have proven effective in amplifying desired genomic DNA fragments from specific filamentous species, the widespread applicability across diverse fungal species remains relatively unexplored. Furthermore, these techniques have not been found to be effective at extracting genomic DNA from the spores of *A*. *niger*, one of the most important industrial filamentous fungi used in biotechnology for producing enzymes and organic acids [12–14]. In contrast to filamentous fungi, direct colony PCR of yeasts has been frequently used for genomic DNA fragment isolations [7, 15, 16]. However, this technique is sometimes hampered by insufficient genomic DNA released into the PCR reaction mix and the presence of PCR inhibitors [17]. As a consequence, contradictory results have been observed and colony PCR efficiency in different yeast species and strains can vary due to different cell wall thickness and cell components [16, 18].

**Fig. 1:**
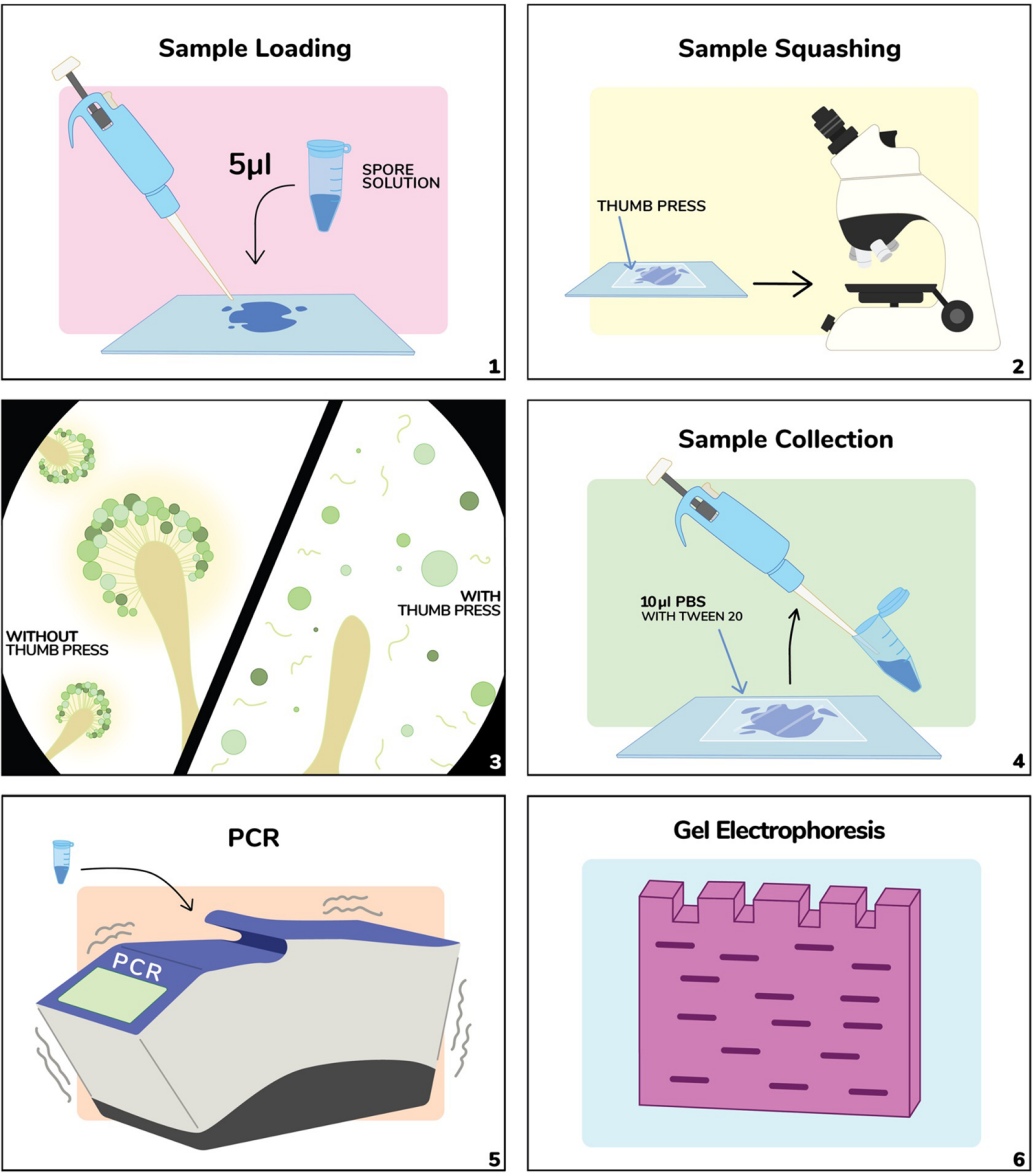
The workflow of squashed spore PCR in fungi

In plant research, a squash technique has been routinely applied during microscopic slide preparation of chromosomal spreading in lily [19, 20] and male meiotic cell division in *Arabidopsis* [21]. As the clearance between glass slide and coverslip is infinitely small, the squashing has been shown to efficiently disrupt the cells. This result prompted us investigate the effectiveness of microscope squashing on disrupting cell walls of various fungal spores. Based on the squash technique, we adapted and developed a novel method for spore PCR which we call Squash-PCR (Sq-PCR, Fig. 1).

In this study, we investigated the effectiveness of Squash-PCR in nine different filamentous fungi and nine different yeast strains. We also explored potential limiting factors of Squash-PCR by focusing on *A*. *niger*.

## Results

### DNA template preparation

The preparation of DNA template for Squash-PCR involved three steps (Fig. 1): (1) loading ~ 5 μL spores onto a microscopy slide, (2) applying pressure to the microscope coverslip to squash the spores, and (3) collecting the DNA sample. By examining the spores on the slide under a microscope before and after the squashing, users can visually assess the levels of the spore cell disruption. Depending on the fungal species, different squash pressures and times may be applied. The squashed spore mixtures can be used for PCR directly or stored at − 20 °C for future use.

**Fig. 2:**
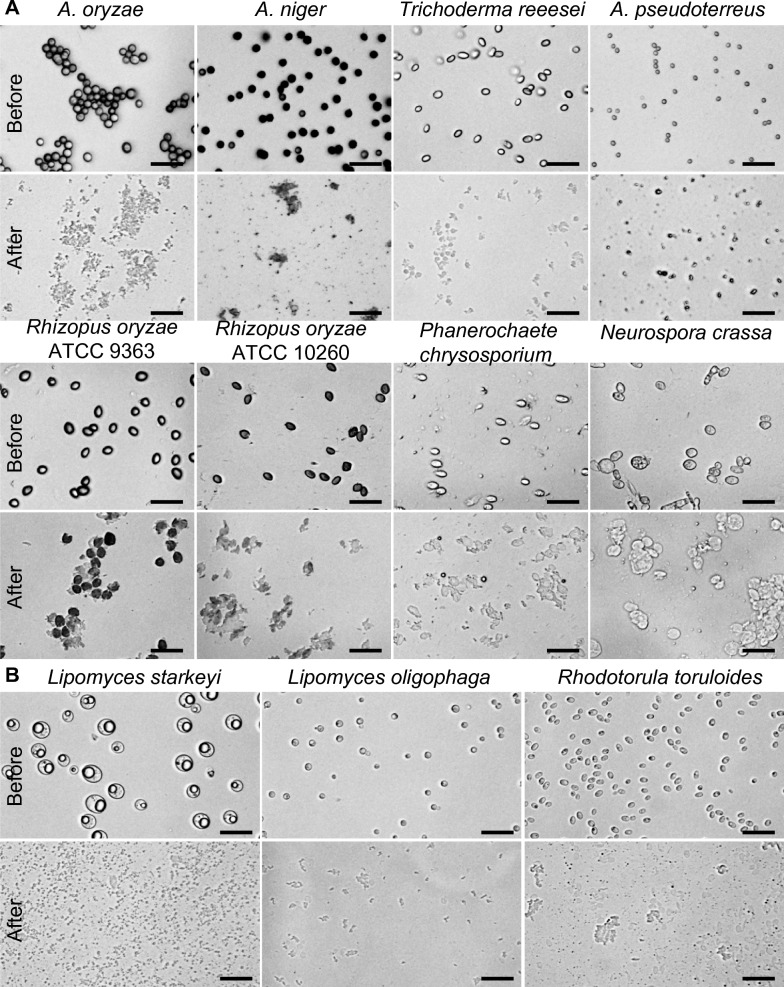
Microscopic view of diverse spores and yeast strains in squash preparations. **A** Microscopic view of eight different spores before and after squash. **B** Microscopic view of three different yeast strains before and after squash. Scale bar = 20 µm

To evaluate the effectiveness of the squashing technique in breaking down cell walls of various fungal spores, we tested nine different filamentous fungi: *Aspergillus oryzae*, *A*. *niger*, *A*. *nidulans, Trichoderma reeesei*, *A. pseudoterreus*, *Rhizopus oryzae*, *Phanerochaete chrysosporium*, *Neurospora crassa* and *Penicillium melinii* (Table 1). We also applied this technique to nine different yeast strains, including: *Lipomyces starkeyi* (NRRL Y-11557), *Lipomyces starkeyi* (ATCC 58680, aka NRRL Y-11557 (mucoid type)) [22], *L*. *doorenjongii*, *L*. *japonicus*, *L*. *oligophage*, *Sacchromyces cerevisiae*, *Kluyveromyces lactis*, *Scheffersomyces stipites* and *Rhodotorula toruloides* (Table 1). Disruption of the spores and yeast cells was confirmed via microscopic examination (Fig. 2). Significant visual differences were observed between samples before and after squashing. Prior to squashing, the fungal spores and yeast cells possessed distinct shapes, sizes, and colors that were easily observed under microscopic imaging (Fig. 2). However, after squashing, only broken cells and cells debris were observed (Fig. 2). These findings indicated that the squashing technique is highly effective and reliable in disrupting various fungal spores and yeast cells.

**Table 1:** Results of spore PCR and colony PCR with and without squash treatments

Fungal species	dsPCR^1^	Sq-PCR^2^	dcPCR^3^	Sq-PCR^4^
*Aspergillus oryaze* ATCC 12892	** − **	** + **	N/A	N/A
*Aspergillus oryaze* ATCC 7252	** − **	** + **	N/A	N/A
*Aspergillus niger* ATCC 11414	** − **	** + **	N/A	N/A
*Aspergillus pseudoterreus* ATCC32359	** − **	** + **	N/A	N/A
*Aspergillus nidulans* ATCC38163	** − **	** + **	N/A	N/A
*Rhizopus oryzae* ATCC 9363	** − **	** + **	N/A	N/A
*Rhizopus oryzae* ATCC 10260	** − **	** + **	N/A	N/A
*Phanerochaete chrysosporium* ATCC 24725	− *	** + **	N/A	N/A
*Neurospora crassa* ATCC MYA-4614	** − **	** + **	N/A	N/A
*Trichoderma reeesei* ATCC13631	− *	** + **	N/A	N/A
*Penicillium melinii* ATCC 13352	** − **	** + **	N/A	N/A
*Lipomyces starkeyi* NRRL Y-11557	N/A	N/A	** + **	** + + **
*Lipomyces starkeyi* ATCC 58680	N/A	N/A	** + **	** + + **
*Lipomyces doorenjongii* NRRL Y-27504	N/A	N/A	** + **	** + + **
*Lipomyces japonicus* CBS 7319	N/A	N/A	** + **	** + + **
*Lipomyces oligophaga* CBS 7107	N/A	N/A	** + **	** + + **
*Sacchromyces cerevisiae* WLP090	N/A	N/A	** + **	** + + **
*Kluyveromyces lactis* NRRL Y-8279	N/A	N/A	** + **	** + + **
*Scheffersomyces stipitis* NRRL Y-11545	N/A	N/A	** + **	** + + **
*Rhodotorula toruloides* NBRC 0880	N/A	N/A	** + **	** + + **

^1^: Direct spore PCR

^2^: Squashed spore PCR

^3^: Direct colony PCR

^4^: Squashed colony PCR

^*^: Faint PCR band

+  + : Improved PCR band

### Squashed spore PCR in diverse fungal strains

**Fig. 3:**
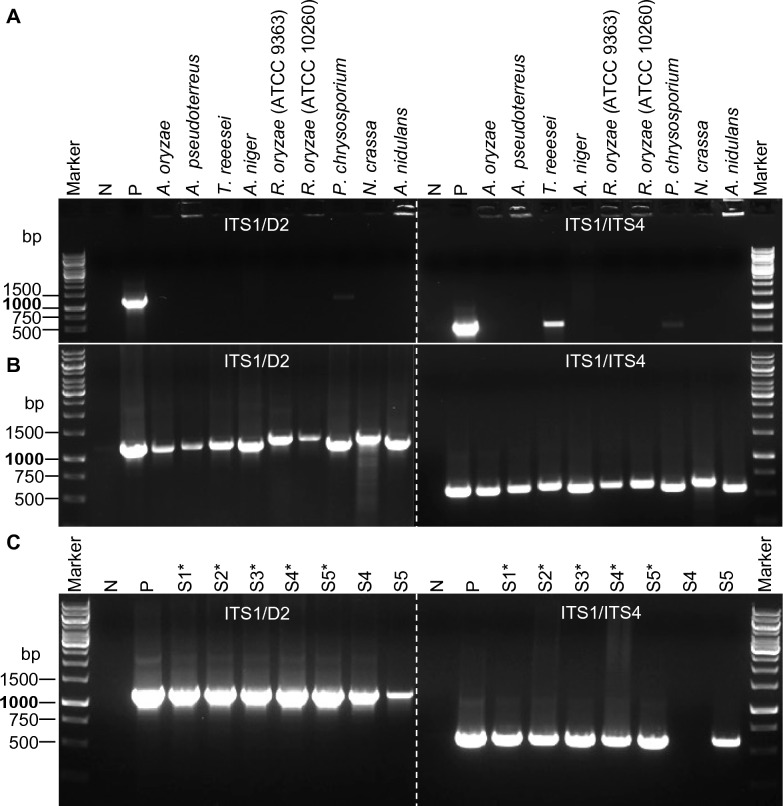
Squashed spore PCR of different filamentous fungi using primers ITS1/D2 and ITS1/ITS4. **A** Direct spore PCR using the supernatant from spore suspensions of nine filamentous. **B** Squashed spore PCR of nine filamentous fungi. **C** Squashed spore PCR of *A. niger*. N, negative control; P, positive control; S1-S5, sample 1–sample 5; *, fivefold dilution

The squashed samples were then collected from the slide by washing with 10 μL of PBS–Tween 20 (v/v, 0.05%) and subjected to PCR. The internal transcribed spacers 1 and 2 (ITS1 and ITS2 regions) and the 28S ribosomal subunit (D1-D2 region) are frequently used as marker regions to characterize fungal biodiversity [23, 24]. Therefore, we used the two primer pairs ITS1/D2 and ITS1/ITS4 for the molecular detection of fungal DNA, which produce expected PCR products sizes of ~ 1.2 kb and ~ 600 bp, respectively. In general, directly using a spore suspension as a DNA template for PCR directly is unlikely to produce high-quality PCR products due to the tough spore cell walls. In our investigation of nine fungal strains, we observed either no or very low amounts of amplified DNA products from the PCR with spore suspension and initial PCR denaturation temperature of 95 °C for 5 min (Fig. 3A). In contrast, using the same PCR program, we were able to amplify desired PCR products in all reactions using squashed spore samples as a DNA template with primer pairs ITS1/D2 and ITS1/ITS4, as shown in Fig. 3B. The genomic DNA of *A*. *niger* extracted via traditional methods were used here as a positive control as well as in all remaining experiments.

After conducting the experiments, it was consistently observed that eight out of nine tested fungal strains produced the expected PCR products using the Sq-PCR method. The only exception was *A*. *niger*, for which it was necessary to dilute the squashed spore samples and use the diluted solution as the template for amplification to improve PCR product yield. To confirm the repeatability of this method, five independent samples (S1 to S5) were prepared via squashing, followed by a fivefold dilution (S1* to S5*, respectively). The PCR products resulting from all five diluted samples (S1* to S5*) were similar to the positive control when using primer pairs ITS1/ITS4 and ITS1/D2. However, lower or no amount of PCR products were generated with the undiluted samples (S4 and S5), as depicted in Fig. 3C. These results highlight the importance of diluting squashed spore samples to achieve effective Squash-PCR in *A*. *niger*. It is worthwhile to note that all tested fungal strains were assessed in a minimum of three biological replicates, which yielded consistent PCR products, indicating the robustness and reproducibility of the Squash-PCR method in fungi.

### Evaluation of relevant parameters impacting squashed spore PCR

**Fig. 4:**
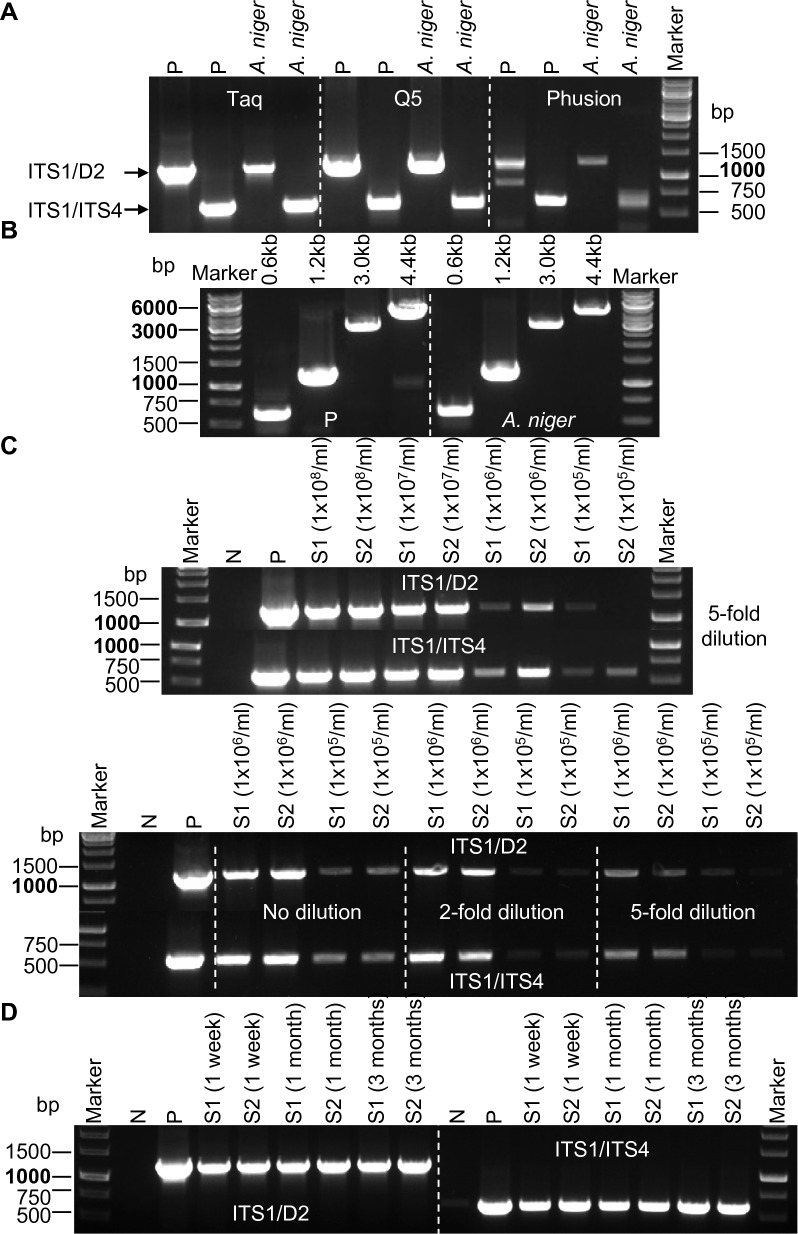
Examination of potential limiting factors that affects Squash-PCR in *A.niger*. **A** PCR to test the efficiency of different DNA polymerases in amplifying PCR products using Squash-PCR with primers ITS1/D2 and ITS1/ITS4. P, positive control; Taq, Taq DNA polymerase; Q5, Q5 High-Fidelity DNA polymerase; Phusion, Phusion High-Fidelity DNA polymerase. **B** The size range of PCR products effectively amplified using Q5 High-Fidelity DNA polymerase in Squash-PCR. P, positive control. **C** The effective range of spore concentration in Squash-PCR. N, negative control; P, positive control. **D** PCR to test the effects of spore age in Squash-PCR. N, negative control; P, positive control

All of the Sq-PCR procedures described above utilized the Taq DNA polymerase. However, for certain activities, such as cloning and sequencing, High-Fidelity DNA polymerase is usually preferred. Therefore, we evaluated two other polymerases, Q5 High-Fidelity DNA Polymerase and Phusion High-Fidelity DNA polymerase in the Squash-PCR of *A*. *niger* using the PCR conditions recommended by the manufacturers (Fig. 4A). Similarly, high-quality PCR products were obtained in the reactions using Taq DNA polymerase and Q5 High-Fidelity DNA polymerase (compared to positive control), whereas relatively poor-quality PCR products (bands) were observed using Phusion High-Fidelity DNA polymerase (Fig. 4A). Therefore, in the tested conditions, the Taq DNA polymerase and Q5 High-Fidelity DNA polymerase appeared to be more robust for *A. niger* Squash-PCR.

To determine the size range of PCR products amplified by Squash-PCR, we amplified a series of PCR products with increasing fragment lengths using Q5 High-Fidelity DNA polymerase in *A*. *niger* (Fig. 4B). In addition to primer pairs of ITS1/ITS4 and ITS1/D2, we included two additional primer pairs, OGY36/OGY38 and OZD2591/OZD2593 to amplify a DNA fragment (jgi|Aspni7|1,145,759| fgenesh1_kg.chr_502_#_298_#_1354_2_EXTC_EXTD) and the *gla1* gene with expected sizes of ~ 3 kb and ~ 4.4 kb, respectively. In the positive control, all the PCR products ranging from 0.6 to 4.4 kb were successfully amplified. Similarly, equivalent PCR products with fragment sizes up to 4.4 kb were also obtained with squashed spore samples after fivefold dilution (compared to the positive control, Fig. 4B), demonstrating the ability of Sq-PCR to efficiently amplify large DNA fragments.

To determine the optimal spore concentration required for Sq-PCR, we conducted an evaluation of *A*. *niger* spore samples with concentrations ranging from 1 × 10^5^ to 1 × 10^8^ spores/mL (Fig. 4C). Two biological replicates were squashed for each spore concentration and each squashed sample was diluted by 5-folds. After PCR, high-quality DNA fragments were obtained in the samples with concentration 1 × 10^7^/mL and 1 × 10^8^/ mL, while faint bands were observed in samples with concentration 1 × 10^5^/mL and 1 × 10^6^/mL. Further investigation revealed that when the spore concentration was at 1 × 10^6^/mL or lower, PCR results of the squashed sample without dilution are superior to those with dilution (Fig. 4C). Overall, samples with spore concentrations between 1 × 10^7^/mL and 1 × 10^8^/mL followed by fivefold dilution after squash were the most optimal for an effective PCR reaction.

In bacteria colony PCR, fresh bacterial plates typically yield better PCR products than older plates. To investigate whether the spore age affects the PCR performance in Sq-PCR, we tested *A. niger* spore samples collected from one week to three months prior to Sq-PCR (Fig. 4D). Comparable PCR products were generated in all samples, regardless of spore age, suggesting that spore age does not have a large effect on Sq-PCR performance in *A. niger*.

### Squashed spore PCR products for Sanger sequencing

**Fig. 5:**
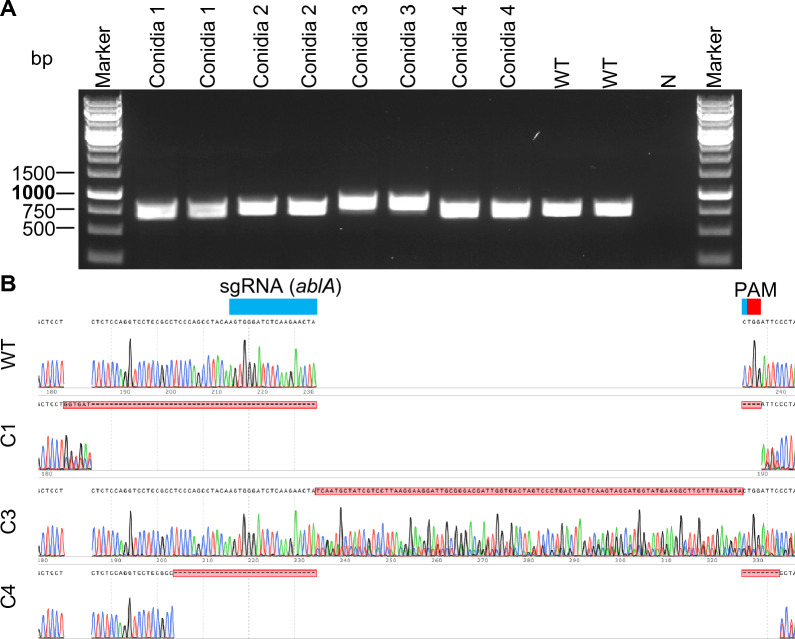
Squash-PCR-mediated rapid screening of transformants in CRISPR-Cas9 genome editing of *A. niger*. **A** Squash-PCR of selected transformants. N, negative control; WT, wild type **B** Sanger sequencing using the PCR products from panel A

CRISPR-Cas technology has been used for genetic editing in various microorganisms. PCR-based genotyping and Sanger sequencing are to date the most frequently used approaches for quick screening and verification of edited genes. Here, we sought to determine whether PCR products generated by Sq-PCR were of sufficient quality for Sanger sequencing. *A. niger* was selected as the test microbe. *A. niger* normally produces black spores with the spore pigment controlled by *albA* gene. The *albA* gene has been commonly used as a selection marker gene because the loss of its function leads to the generation of white spores [25, 26]. CRISRP-Cas9 mediated genome editing targeting *albA* gene was performed, and four conidia of transformants were selected for Sq-PCR with the wild type (WT) *A*. *niger* as a control. All selected conidia as well as the WT generated the expected PCR products around 800 bp (Fig. 5A). Deletion/insertion events caused by CRISPR-Cas9 were successfully detected in 3 out of 4 transformed conidia through Sanger sequencing with the purified PCR products. Specifically, a combined insertion/deletion event (5 bp and 54 bp), an insertion event (93 bp) and a deletion event (39 bp) were confirmed by Sanger sequencing in conidia #1, #3 and #4, respectively (Fig. 5B). The editing profile of conidium #2 failed to read due to a poor Sanger sequencing quality.

### Effects of pigments on squashed spore PCR

**Fig. 6:**
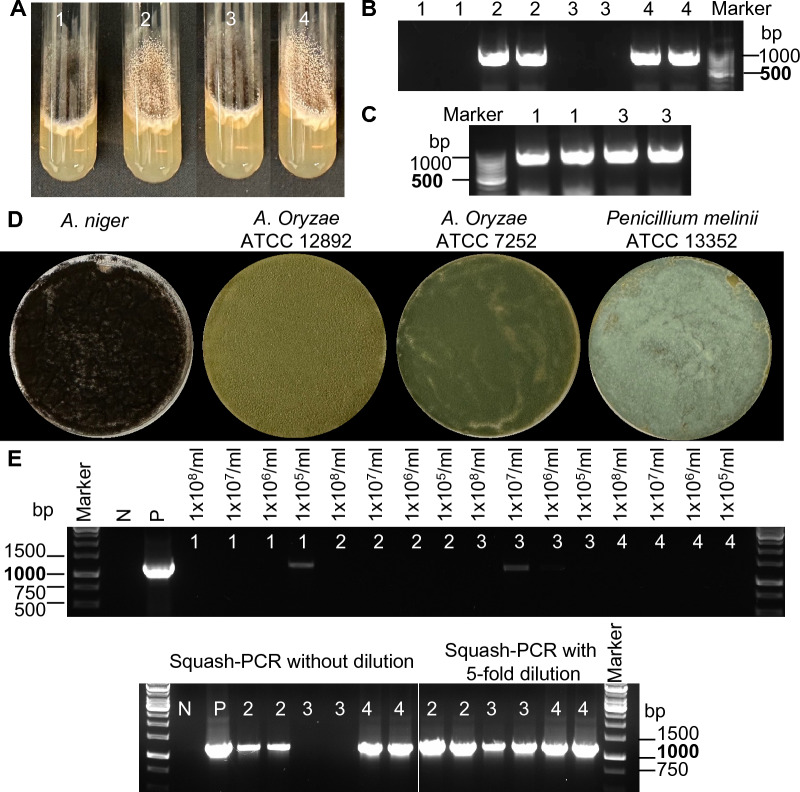
Characterization of the effects of pigments on Squash-PCR in fungi. **A** The phenotype of *albA* transformants. **B** Squash-PCR using black spores (1 and 3) and white spores (2 and 4) without dilution of template, with primers OGY11/ OGY12. **C** Squash-PCR using black spores (1 and 3) with fivefold dilution of template DNA, with primers OGY11/ OGY12. **D** Spore pictures of *A*. *niger*, *A*. *Oryzae* ATCC 12892,* A*. *Oryzae* ATCC 7252 and *Penicillium melinii* ATCC 13352. **E** Spore PCR using the spores from **D**, with primers ITS1/D2. N, negative control; P, positive control; 1, *A*. *niger*; 2, *A*. *Oryzae* ATCC 12892; 3, *A*. *Oryzae* ATCC 7252; 4, *Penicillium melinii* ATCC 13352. Two technique replicates were performed for Squash-PCR

Since it has been reported that melanin inhibits PCR amplification [27], we investigated the impact of melanin on PCR amplification by analyzing the black spores and white spores obtained from CRISRP-Cas9 gene editing of *albA* in *A*. *niger*. Two black spores and two white spores were simultaneously examined through Squash-PCR (Fig. 6A). Interestingly, high-quality genomic DNA PCR fragments were produced with the white spores from the *albA* mutant strain when using the squashed samples without dilution. However, no PCR products were detected with those squashed samples of the black spores (Fig. 6B). As expected, with a fivefold dilution of the crushed sample of black spores, targeted PCR products were obtained via Squash-PCR (Fig. 6C). Therefore, it indicates that Sq-PCR was also severely affected by the melanin in *A*. *niger* spores.

The effects of spore pigments from other fungal strains on Sq-PCR were next evaluated. Three filamentous fungal strains *A*. *Oryzae* (ATCC 12892 and ATCC 7252) and *Penicillium melinii* (ATCC 13352) that contain green pigments were selected for testing (Fig. 6D). No or very low amount of PCR products were obtained with oligo pair of ITS1/D2 when the intact spores were used (Fig. 6E). The squashed spore samples with or without fivefold dilution were simultaneously compared. Samples with no dilution of squashed spores resulted in the expected PCR products in strains *A*. *oryzae*-ATCC12892 and *P. melinii*, but not in *A*. *oryzae-*ATCC7252. In contrast, high-quality PCR fragments were produced in all three strains with fivefold dilution of the squashed spore samples. The PCR product band quality of *A*. *oryzae*-ATCC12892 were significantly improved by fivefold dilution, but no distinguishable difference was observed in *P. melinii* before and after dilution. In conclusion, our findings suggest that spore pigments inhibit Sq-PCR not only in *A*. *niger* but also in *A*. *oryzae*. Dilution of the squashed spore samples can significantly improve the production of selected PCR fragments in filamentous fungal strains containing spore pigments.

### Squashed colony PCR in diverse yeast strains

**Fig. 7:**
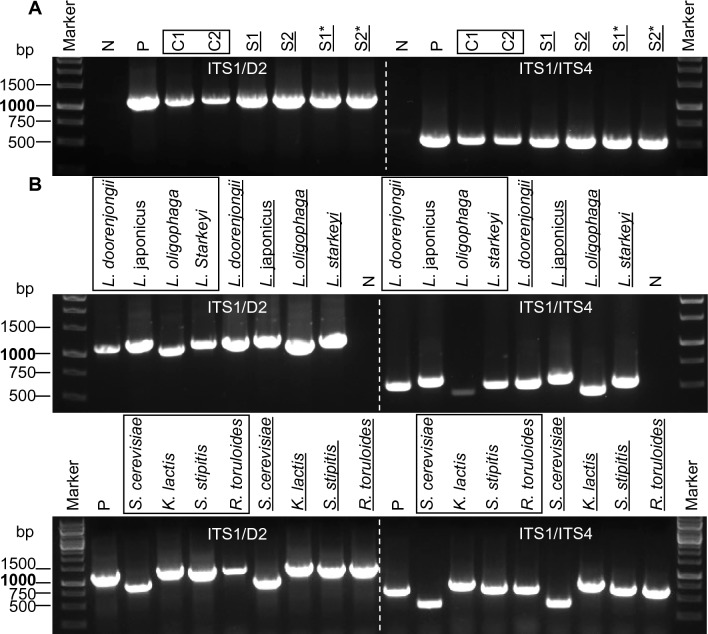
Squashed colony PCR of different yeast strains with primers ITS1/D2 and ITS1/ITS4. **A** Squashed colony PCR of *Lipomyces starkeyi* (NRRL Y-11557). **B** Squashed colony PCR of eight different yeast strains. The boxes indicate direct colony PCR. The underlines indicate squashed colony PCR. *Lipomyces starkeyi* (ATCC 58680); N, negative control; P, positive control; C1 and C2, colony 1 and colony 2; S1 and S2, sample 1 and sample 2 from colony 1 and colony 2, respectively; *, fivefold dilution

Given that dilution of squashed spore sample resulted in an improved PCR reaction compared to the undiluted one (FigS. 3C and 6E), colony PCR of *L*. *starkeyi* was evaluated for its effectiveness with colony samples obtained from the intact (C1 and C2) or squashed (S1 and S2) cells as well as fivefold diluted squashed samples (S1* and S2*) (Fig. 7A). High quality PCR products were observed in all reactions after PCR with no distinguishable difference between diluted and undiluted squashed colony samples, indicating the dilution may not be necessary to obtain optimal PCR results in yeast. In comparison to colony PCR with intact cells, more PCR products were achieved in squashed colony PCR both with and without dilution (Fig. 7A). To further investigate the applicability of squashed colony PCR, we tested Sq-PCR in additional eight different yeast strains of various industrial importance. Sharp bands of PCR products were achieved in almost all PCR reactions using direct colony samples and squashed colony samples (Fig. 7B). Comparatively broader PCR bands were observed in the squashed colony samples, in contrast to the direct colony samples (Fig. 7A, B), suggesting that Squash-PCR is a superior method for colony PCR across diverse yeast strains.

## Discussion

Several methods of spore PCR have been reported, but they have not been shown to be widely applicable for molecular characterization and engineering of various fungal species due to poor yield and repeatability of PCR products. Here, we demonstrate that spore squashing is an effective experimental treatment for inducing spore disruption and DNA release in multiple fungal and yeast species. Sq-PCR has an added benefit of being a quick and effective means of breaking open cells without the need for hazardous reagents used in traditional methods. Furthermore, the developed Sq-PCR method can be used for spore PCR of *A*. *niger*, which has not been previously reported (to our knowledge). Our results have shown that several different polymerases were all able to reliably yield PCR products, but that the Phusion High-Fidelity polymerase resulted in poorer quality PCR products. Although it should be noted that we did not attempt to optimize conditions using the Phusion polymerase, and better results may be achievable with optimized parameters. The use of Q5 polymerase allowed for effective amplification of up to 4.4 kb DNA fragments in *A*. *niger*. This demonstrated that genes of varying size ranges can be effectively amplified through Sq-PCR. Furthermore, our results demonstrated that spore concentrations within the range of 1 × 10^7^/mL to 1 × 10^8^/mL exhibited significantly higher PCR product yields compared to lower concentrations. This finding aligns with the thermal-shock method, where PCR amplicons were detected in the spore supernatant across a concentration range of 7.8 × 10^5^/mL to 8 × 10^8^/mL of spores [9]. Finally, we have also shown that spore age had limited effect on the quality of PCR products, in contrast to colony PCR using bacteria and yeast where fresh colonies typically yield better results [15, 28, 29]. The squashed spore samples can be stored in freezer at least three months without affecting PCR quality.

Compared to other fungal strains, spore PCR proved to be more challenging in *A*. *niger*, which is consistent with previous results [9]. Although we were able to obtain expected PCR products using the squashed spore samples of *A. niger* with spore concentrations ranging between 1 × 10^7^/mL and 1 × 10^8^/mL, poor repeatability and variable results were frequent occurrences. Based on reports that the presence of PCR inhibitors from yeast cells might inhibit colony PCR [17] and that PCR can be hampered by an excess of spores [10], we hypothesized that the large amount of cell components from *A*. *niger* spores may contain excessive PCR inhibitors as well. Thus, reducing the amount of PCR inhibitor of squashed spore sample was critical. By diluting the DNA template of the squashed spores, the dosage of PCR inhibitor per unit volume can be reduced and may potentially result in improved PCR products. Indeed, enhanced PCR products were achieved using samples with fivefold dilution after squashing spores with concentrations ranging between 1 × 10^7^/mL and 1 × 10^8^/mL. In contrast, PCR bands with low yields were seen for *A*. *niger* spore samples with lower concentrations (1 × 10^5^/mL to 1 × 10^6^/mL), along with fivefold dilution after squashing spores. This may be due to the concentration of genomic DNA present being below the effective template threshold for PCR. Maintaining the balance between low PCR inhibitor concentrations and keeping the genomic DNA concentration above the effective template threshold is the key for optimizing squashed spore PCR. Therefore, no dilution is preferred to generate greater yields of PCR products for *A*. *niger* with spore concentrations below 1 × 10^6^/mL.

It should be noted that the outcome observed in *A*. *niger* were not observed among the other filamentous fungal strains investigated, suggesting the possibility that *A. niger* spores contain an excessive amount of certain PCR inhibitors. Many organic substances such as bile salts, urea, phenol, ethanol, polysaccharides, sodium dodecyl sulfate (SDS) and various proteins including collagen, myoglobin, lactoferrin, and proteinases are currently recognized as PCR inhibitors [30]. A distinctive feature of *A*. *niger* is black conidiospores due to the production of melanin. Moreover, melanin has been identified as a potent PCR inhibitor and it can suppress PCR results even at very small amounts [27, 31]. Therefore, we examined the effect of melanin on Sq-PCR by examining a mutant strain *albA*, that produces white spores. Optimal PCR products were obtained via Sq-PCR in white spores but not in black spores under the same conditions, indicating that melanin is a primary inhibitor of spore PCR in *A*. *niger*. Similar results were also observed in two *A*. *oryzae* (ATCC 12892 and ATCC 7252) strains which contained green pigments. Remarkably, our findings suggest that fungal species with darker pigmentation (*A*. *niger* and ATCC 7252) exhibited reduced PCR amplification compared to those with relatively lighter pigmentation (*Penicillium melinii* and ATCC 12892). Therefore, it’s clear that pigments act as major PCR inhibitors in Sq-PCR though other potential PCR inhibitors, such as polysaccharides and hydrophobins components of spore cell walls [32] may also play a role in suboptimal PCR reactions. In this study, we demonstrated that reducing PCR inhibitors by diluting the squashed spore sample was a simple and effective method for obtaining PCR products in fungal strains possessing pigments. Alternatively, adding BSA to the DNA template has also been used to neutralize the inhibition of melanin in PCR [27].

Recently, the CRISPR-Cas mediated genome editing has been used for genetic engineering of various fungi, including *Aspergillus nidulans*, *A. aculeatus*, *A. niger*, *A. carbonarius*, *A. luchuensis*, *A. brasiliensis*, *A. oryzae*, *Candida lusitaniae* and *Trichoderma reesei* [33–36]. Screening of transformants and identification of edited events extensively relies on PCR-based approaches that use genomic DNA as a template. As mentioned earlier, the application of Sq-PCR enabled us to bypass these time-consuming procedures and identify the CRISPR-Cas edited events much quicker and easier than the traditional ones. In addition to mutant screening, we have also employed Sq-PCR to generate DNA fragments for vector cloning (Additional file 1: Fig. S1). The Sq-PCR-generated DNA fragments proved to be highly effective in downstream vector assemble. Squash-PCR drastically reduces manipulation and template preparation time and therefore decreases the test turnaround time and accelerate the genetic engineering of *A. niger* strains.

**Fig. 8:**
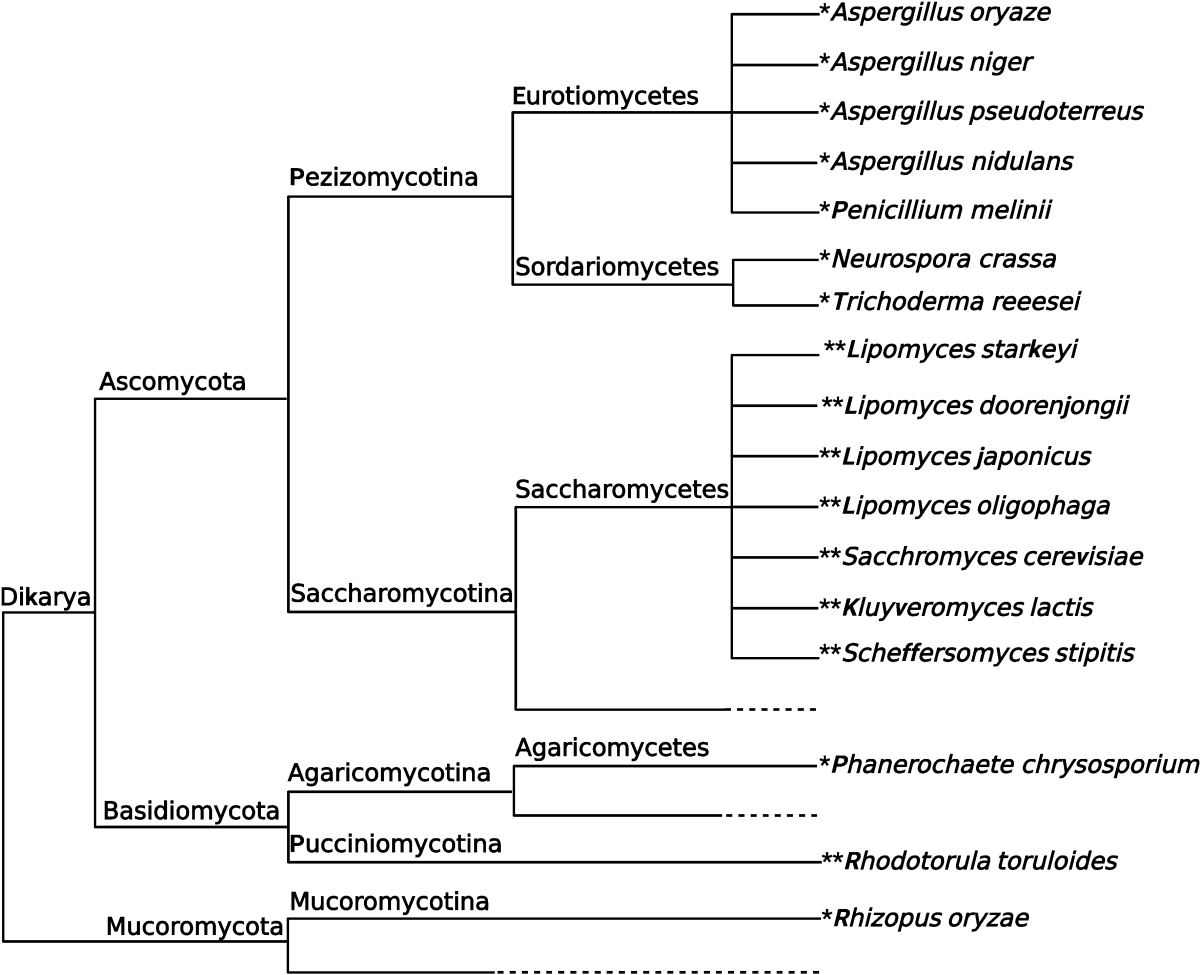
Simplified phylogenetic tree of fungi used for spore and colony PCR. *, fungal species used for spore PCR; **, yeast species used for colony PCR. This figure is adapted from Grigoriev et al.[38]

Furthermore, we also found that the colony PCR can be enhanced dramatically in squashed colony PCR in comparison with those in direct colony PCR. When low amplification efficiencies are observed in colony PCR of yeast colonies, squashed colony PCR would be an alternative solution. For instance, the deletion of multiple *N*-mannosyltransferases in *Saccharomyces cerevisiae* leads to strains that are no longer amenable to analysis using conventional PCR on yeast colonies [16]. A total of eleven fungal strains representing nine fungal species, as well as nine yeast strains representing eight yeast species, were subjected to Squash-PCR, resulting in a remarkable 100% success rate (Fig. 8).

While Squash-PCR is recognized as an easy and reliable method, further improvements are necessary to tackle larger-scale projects and process a substantial number of samples with ease. Automation of the squashing process can streamline sample preparation and increase the number of samples processed simultaneously. This can be achieved using robotic liquid handling systems or specialized squashing devices. Such a system provides a platform for efficient DNA extraction in 96-well plate for downstream PCR amplification. The ability to process multiple samples coupled with automation will advance the metabolic engineering progress in various fields.

## Conclusion

Overall, the Squash-PCR method developed in this research is a simple, fast, and efficient PCR screening technique that does not involve toxic reagents usage. It can be applied to various microorganisms, regardless of their cell wall structure complexity, as the working mechanism only involves mechanically breaking the cells. With rapid advancements in multi-omics analysis and synthetic biology tools, such as the CRISPR-Cas system, screening requirements are scaling up as more genetically engineered strains need to undergo PCR screening before initiating the test and learn cycle. This method is expected to accelerate the Design-Build-Test-Learn (DBTL) cycle in biomanufacturing development.

## Materials and methods

### Strains

The fungal species and yeast strains used for squashed spore/colony PCR are listed in Table 1.

### Squash preparation

Squash preparations were prepared as follows, 1) load 5 μL spore solution onto a glass slide and cover it with a coverslip. 2) Place the thumb on the coverslip and press firmly while moving the coverslip horizontally. 3) lift the coverslip and add 10 μL PBS–Tween 20 (v/v, 0.05%) solution. 4) collect the spore solution using a pipette and dilute it with 10 μL or more PBS–Tween 20 as needed. For more detailed instruction, please refer to Additional file 2: Video S1.

### PCR with taq DNA polymerase

In this PCR protocol, GoTaq green master mixes (Promega) were used following PCR conditions specified as below. 1 μL of spore solution was used as a template for 20 μL total PCR reaction volume with the PCR cycling conditions: 95 °C for 2 min, 35 cycles of 95 °C for 30 s, 55 °C for 30 s, and 72 °C for 1 min, with the final elongation step at 72 °C for 5 min.

### PCR with Q5 DNA polymerase

In this PCR protocol, Q5 High-Fidelity 2X master mix (New England BioLabs) were used following PCR conditions specified as below. 1 μL of spore solution was used as a template for 20 μL total PCR reaction volume with the PCR cycling conditions: 98 °C for 30 s, 35 cycles of 98 °C for 10 s, 65 °C for 30 s, and 72 °C for 20 s ~ 2 min based on different PCR size (20–30 s/kb), with the final elongation step at 72 °C for 2 min.

### PCR with Phusion polymerase

In this PCR protocol, Phusion High-Fidelity DNA Polymerases (Thermo Scientific) were used following PCR conditions specified as below. 1 μL of spore solution was used as a template for 20 μL total PCR reaction volume with the PCR cycling conditions: 98 °C for 1 min, 35 cycles of 98 °C for 10 s, 65 °C for 30 s, and 72 °C for 30 s, with the final elongation step at 72 °C for 2 min.

### Primers for PCR

All primers used for spore and colony PCR are listed in Additional file 3: Table S1.

### Microscopy

Microscopic examination was carried out to check the status of spores before and after squashing, using a Zeiss laser microdissection microscope (Zeiss PALM microbeam-Laser microdissection).

### Spore counting

The number of spores were counted using a hemocytometer under a microscope. Spore concentrations were determined as described previously [37].

### Gel purification

The PCR products were purified using the MinElute gel extraction kit (QIAGEN) and quantified using a NanoDrop.

### Sanger sequencing

Purified PCR products were sequenced by GENEWIZ.

## Supplementary Information

**Additional file 1: Figure S1.** Squash-PCR-based DNA fragment amplification for vector cloning. Squash-PCR was used to amplify DNA fragment 1 and 4 with primers OZD3062/OZD3063 and OZD3065/OZD3066, respectively. Fragments 2 and 3 were amplified using plasmid DNA as the template.

**Additional file 2: Video S1.** Squash spore preparation.

**Additional file 3: Table S1.** The oligos used for spore PCR and colony PCR.

## Data Availability

All data generated or analyzed during this study are provided within this published article and its supplementary information files.

## References

[1] Frisvad JC, Andersen B, Thrane U (2008). The use of secondary metabolite profiling in chemotaxonomy of filamentous fungi. Mycol Res.

[2] Frisvad JC, Møller LLH, Larsen TO, Kumar R, Arnau J (2018). Safety of the fungal workhorses of industrial biotechnology: update on the mycotoxin and secondary metabolite potential of *Aspergillus **niger*, *Aspergillus **oryzae*, and *Trichoderma **reesei*. Appl Microbiol Biotechnol.

[3] Wösten HAB (2019). Filamentous fungi for the production of enzymes, chemicals and materials. Curr Opin Biotechnol.

[4] Khan MI, Shin JH, Kim JD (2018). The promising future of microalgae: current status, challenges, and optimization of a sustainable and renewable industry for biofuels, feed, and other products. Microb Cell Fact.

[5] Watanabe M, Lee K, Goto K, Kumagai S, Sugita-Konishi Y (2010). Rapid and effective DNA extraction method with bead grinding for a large amount of fungal DNA. J Food Prot.

[6] Karakousis A, Tan L, Ellis D, Alexiou H, Wormald PJ (2006). An assessment of the efficiency of fungal DNA extraction methods for maximizing the detection of medically important fungi using PCR. J Microbiol Methods.

[7] Packeiser H, Lim C, Balagurunathan B, Wu J, Zhao H (2013). An extremely simple and effective colony PCR procedure for bacteria, yeasts, and microalgae. Appl Biochem Biotechnol.

[8] Ferreira A, Glass N, Louise N: PCR from fungal spores after microwave treatment. Fungal Genetics Newsletter 1996:25–26.

[9] Fraczek MG, Zhao C, Dineen L, Lebedinec R, Bowyer P, Bromley M, Delneri D (2019). Fast and reliable PCR amplification from *Aspergillus fumigatus* spore suspension without traditional DNA extraction. Curr Protoc Microbiol.

[10] Xu J-R, Hamer JE (1995). Assessment of *Magnaporthe** grisea* mating type by spore PCR. Fungal Genet Newsletter.

[11] Alshahni MM, Makimura K, Yamada T, Satoh K, Ishihara Y, Takatori K, Sawada T (2009). Direct colony PCR of several medically important fungi using Ampdirect plus. Jpn J Infect Dis.

[12] Frisvad JC, Larsen TO, Thrane U, Meijer M, Varga J, Samson RA, Nielsen KF (2011). Fumonisin and ochratoxin production in industrial *Aspergillus **niger* strains. PLoS ONE.

[13] Niu J, Arentshorst M, Nair PD, Dai Z, Baker SE, Frisvad JC, Nielsen KF, Punt PJ, Ram AF: Identification of a classical mutant in the industrial host *Aspergillus niger* by systems genetics: laea is required for citric acid production and regulates the formation of some secondary metabolites. G3 (Bethesda) 2015, 6(1):193–204. 10.1534/g3.115.024067 PMC4704718 26566947

[14] Pel HJ, de Winde JH, Archer DB, Dyer PS, Hofmann G, Schaap PJ, Turner G, de Vries RP, Albang R, Albermann K (2007). Genome sequencing and analysis of the versatile cell factory *Aspergillus **niger* CBS 513.88. Nat Biotechnol.

[15] Mirhendi H, Diba K, Rezaei A, Jalalizand N, Hosseinpur L, Khodadadi H (2007). Colony PCR is a rapid and sensitive method for DNA amplification in yeasts. Iran J Public Health.

[16] Bonnet C, Rigaud C, Chanteclaire E, Blandais C, Tassy-Freches E, Arico C, Javaud C (2013). PCR on yeast colonies: an improved method for glyco-engineered *Saccharomyces cerevisiae*. BMC Res Notes.

[17] Azevedo F, Pereira H, Johansson B (2017). Colony PCR. Methods Mol Biol.

[18] Bzducha-Wróbel A, Błażejak S, Tkacz K (2012). Cell wall structure of selected yeast species as a factor of magnesium binding ability. Eur Food Res Technol.

[19] Zhou S, Yuan G, Xu P, Gong H (2014). Study on lily introgression breeding using allotriploids as maternal parents in interploid hybridizations. Breed Sci.

[20] Zhou S, Tan X, Fang L, Jian J, Xu P, Yuan G (2013). Study of the female fertility of an odd-tetraploid of lilium and its potential breeding significance. J Am Soc Hort Sci.

[21] Yuan G, Ahootapeh BH, Komaki S, Schnittger A, Lillo C, De Storme N, Geelen D (2018). PROTEIN PHOSHATASE 2A B’α and β maintain centromeric sister chromatid cohesion during meiosis in arabidopsis. Plant Physiol.

[22] Dai Z, Deng S, Culley DE, Bruno KS, Magnuson JK (2017). Agrobacterium tumefaciens-mediated transformation of oleaginous yeast Lipomyces species. Appl Microbiol Biotechnol.

[23] Hinrikson HP, Hurst SF, de Aguirre L, Morrison CJ (2005). Molecular methods for the identification of Aspergillus species. Med Mycol.

[24] Bellemain E, Carlsen T, Brochmann C, Coissac E, Taberlet P, Kauserud H (2010). ITS as an environmental DNA barcode for fungi: an in silico approach reveals potential PCR biases. BMC Microbiol.

[25] Li C, Zhou J, Rao S, Du G, Liu S (2021). Visualized Multigene Editing System for Aspergillus niger. ACS Synth Biol.

[26] Vanegas KG, Jarczynska ZD, Strucko T, Mortensen UH (2019). Cpf1 enables fast and efficient genome editing in Aspergilli. Fungal Biol Biotechnol.

[27] Eckhart L, Bach J, Ban J, Tschachler E (2000). Melanin binds reversibly to thermostable DNA polymerase and inhibits its activity. Biochem Biophys Res Commun.

[28] Shahmoradi M, Faridifar P, Shapouri R, Mousavi SF, Ezzedin M, Mirzaei B (2019). Determining the biofilm forming gene profile of Staphylococcus aureus clinical isolates via multiplex colony PCR method. Rep Biochem Mol Biol.

[29] Murray JM, Watson AT, Carr AM (2016). Colony polymerase chain reaction with *Schizosaccharomyces** pombe*. Cold Spring Harb Protoc.

[30] Schrader C, Schielke A, Ellerbroek L, Johne R (2012). PCR inhibitors—occurrence, properties and removal. J Appl Microbiol.

[31] Frouin E, Maudelonde T, Senal R, Larrieux M, Costes V, Godreuil S, Vendrell JA, Solassol J (2016). Comparative methods to improve the detection of BRAF V600 mutations in highly pigmented melanoma specimens. PLoS ONE.

[32] Cortesão M, de Haas A, Unterbusch R, Fujimori A, Schütze T, Meyer V, Moeller R (2020). *Aspergillus **niger* spores are highly resistant to space radiation. Front Microbiol.

[33] Norton EL, Sherwood RK, Bennett RJ (2017). Development of a CRISPR-Cas9 system for efficient genome editing of *Candida **lusitaniae*. MSphere.

[34] Katayama T, Tanaka Y, Okabe T, Nakamura H, Fujii W, Kitamoto K (2016). Maruyama J-i: Development of a genome editing technique using the CRISPR/Cas9 system in the industrial filamentous fungus *Aspergillus **oryzae*. Biotech Lett.

[35] Nødvig CS, Nielsen JB, Kogle ME, Mortensen UH (2015). A CRISPR-Cas9 system for genetic engineering of filamentous fungi. PLoS ONE.

[36] Liu R, Chen L, Jiang Y, Zhou Z, Zou G (2015). Efficient genome editing in filamentous fungus Trichoderma reesei using the CRISPR/Cas9 system. Cell Discovery.

[37] Thesseling FA, Bircham PW, Mertens S, Voordeckers K, Verstrepen KJ (2019). A hands-on guide to brewing and analyzing beer in the laboratory. Curr Protoc Microbiol.

[38] Grigoriev IV, Cullen D, Goodwin SB, Hibbett D, Jeffries TW, Kubicek CP, Kuske C, Magnuson JK, Martin F, Spatafora JW (2011). Fueling the future with fungal genomics. Mycology.

