# Supplementary figures and images for: Rapid and robust squashed spore/colony PCR of industrially important fungi
Source: Fungal Biol Biotechnol. 2023 Jul 8;10:15. doi: 10.1186/s40694-023-00163-0 (PMC10329332; doi:10.1186/s40694-023-00163-0)

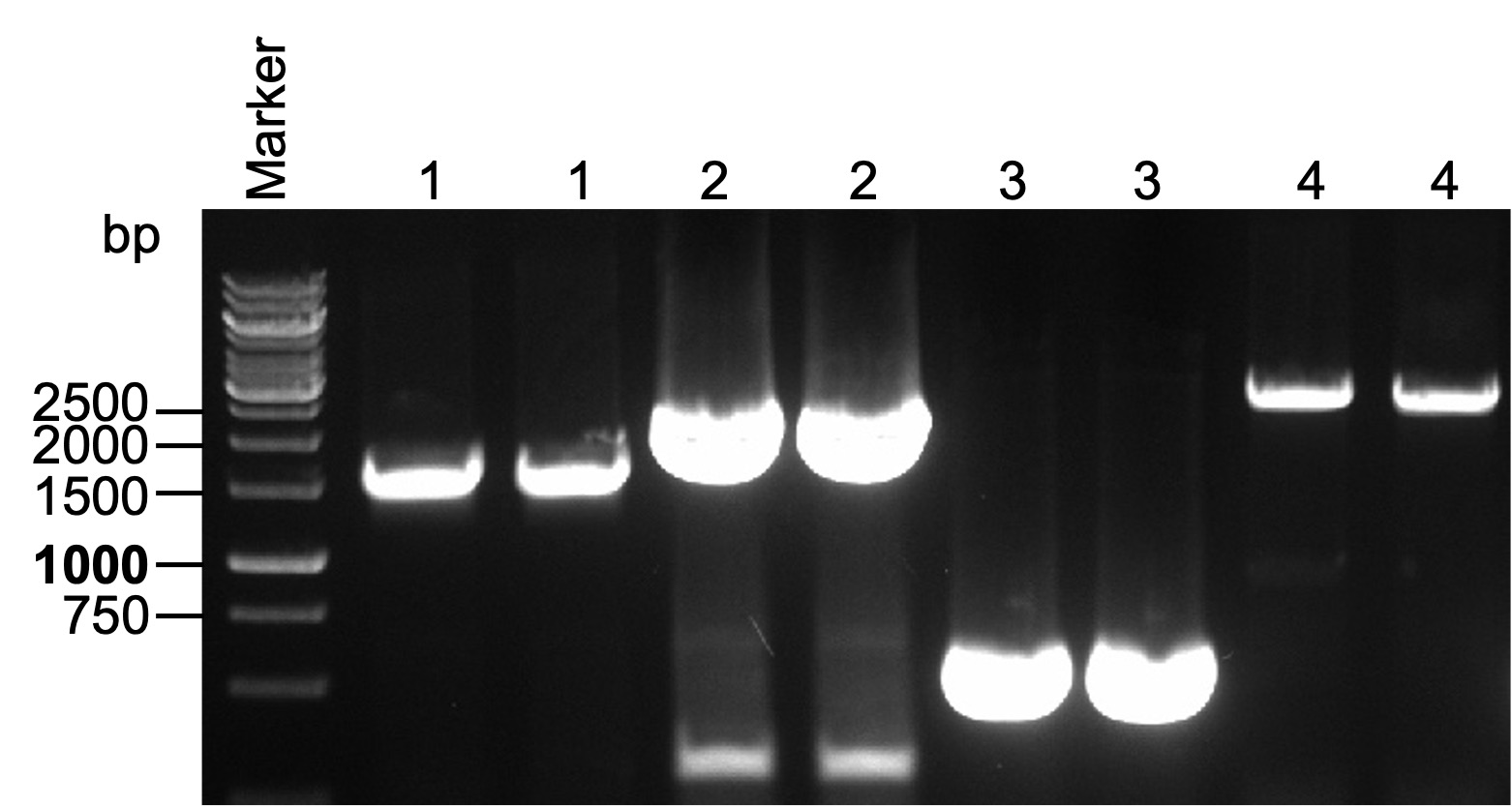

Supplement: Supplementary file 1 — Additional file 1: Figure S1. Squash-PCR-based DNA fragment amplification for vector cloning. Squash-PCR was used to amplify DNA fragment 1 and 4 with primers OZD3062/OZD3063 and OZD3065/OZD3066, respectively. Fragments 2 and 3 were amplified using plasmid DNA as the template. [file 40694_2023_163_MOESM1_ESM.jpg]
