# Supplementary material for: Rapid and robust squashed spore/colony PCR of industrially important fungi
Source: Fungal Biol Biotechnol. 2023 Jul 8;10:15. doi: 10.1186/s40694-023-00163-0 (PMC10329332; doi:10.1186/s40694-023-00163-0)
Supplement: Supplementary file 3 — Additional file 3: Table S1. The oligos used for spore PCR and colony PCR. [file 40694_2023_163_MOESM3_ESM.docx]

**Additional file 3: Table S1. The oligos used for spore PCR and colony PCR**

| **Name** | **Sequence** |
| --- | --- |
| ITS1 | TCCGTAGGTGAACCTGCGG |
| D2 | TTGGTCCGTGTTTCAAGACG |
| ITS4 | TCCTCCGCTTATTGATATGC |
| OGY11 | GACCAA TGACAAGACTCTGTGGGT |
| OGY12 | TCTTCTTCCCCTCCGCAGTGAC |
| OGY36 | ATGCGTCATCATCTGCTCAG |
| OGY38 | CCAGAGATCCTAGACGACAC |
| OZD2591 | CGAGAGCAGCTTGAAGAACT |
| OZD2593 | CTTGATTCTCGCCTGCGTAT |
| OZD3062 | CTTCATCAAGATGTCGTTCCGATCTCTTCTCGC |
| OZD3063 | GCCGAAGACCTCATTGGTCGTCCGGGCTGAGTG |
| OZD3065 | CGAGGTCGACGGTATCGATAATCCGAACTCCAACCGGGGG |
| OZD3066 | GCCGAAGACCTCATTGGTCGTCCGGGCTGAG |
